# Comparison of insulin sensitivity between healthy neonatal foals and horses using minimal model analysis

**DOI:** 10.1371/journal.pone.0262584

**Published:** 2022-01-14

**Authors:** Hannah M. Kinsella, Laura D. Hostnik, Hailey A. Snyder, Sarah E. Mazur, Ahmed M. Kamr, Teresa A. Burns, John C. Mossbarger, Ramiro E. Toribio

**Affiliations:** 1 College of Veterinary Medicine, The Ohio State University, Columbus, Ohio, United States of America; 2 Faculty of Veterinary Medicine, University of Sadat City, Sadat City, Egypt; 3 Midland Acres Inc, Bloomingburg, Ohio, United States of America; Medical University of Vienna, AUSTRIA

## Abstract

The equine neonate is considered to have impaired glucose tolerance due to delayed maturation of the pancreatic endocrine system. Few studies have investigated insulin sensitivity in newborn foals using dynamic testing methods. The objective of this study was to assess insulin sensitivity by comparing the insulin-modified frequently sampled intravenous glucose tolerance test (I-FSIGTT) between neonatal foals and adult horses. This study was performed on healthy neonatal foals (n = 12), 24 to 60 hours of age, and horses (n = 8), 3 to 14 years of age using dextrose (300 mg/kg IV) and insulin (0.02 IU/kg IV). Insulin sensitivity (SI), acute insulin response to glucose (AIRg), glucose effectiveness (Sg), and disposition index (DI) were calculated using minimal model analysis. Proxy measurements were calculated using fasting insulin and glucose concentrations. Nonparametric statistical methods were used for analysis and reported as median and interquartile range (IQR). SI was significantly higher in foals (18.3 L·min^-1^· μIU^-1^ [13.4–28.4]) compared to horses (0.9 L·min^-1^· μIU^-1^ [0.5–1.1]); (p < 0.0001). DI was higher in foals (12 × 10^3^ [8 × 10^3^−14 × 10^3^]) compared to horses (4 × 10^2^ [2 × 10^2^−7 × 10^2^]); (p < 0.0001). AIRg and Sg were not different between foals and horses. The modified insulin to glucose ratio (MIRG) was lower in foals (1.72 μIU_insulin_^2^/10·L·mg_glucose_ [1.43–2.68]) compared to horses (3.91 μIU _insulin_^2^/10·L·mg_glucose_ [2.57–7.89]); (p = 0.009). The homeostasis model assessment of beta cell function (HOMA-BC%) was higher in horses (78.4% [43–116]) compared to foals (23.2% [17.8–42.2]); (p = 0.0096). Our results suggest that healthy neonatal foals are insulin sensitive in the first days of life, which contradicts current literature regarding the equine neonate. Newborn foals may be more insulin sensitive immediately after birth as an evolutionary adaptation to conserve energy during the transition to extrauterine life.

## 1. Introduction

At birth, the neonatal foal undergoes numerous intense physiologic changes during the transition from intra-uterine to extra-uterine life. As neonates transition from parenteral nutrition via the placenta to enteral nutrition immediately after birth, maturation of the endocrine system is of utmost importance, as the endocrine pancreas must assume a glucoregulatory role in order to maintain euglycemia [1, 2]. The endocrine pancreas of the equine fetus is functional prior to parturition, as pancreatic alpha and beta cells are responsive to intra-fetal infusion of amino acids and glucose [3, 4]. The maturation of the endocrine pancreas continues into the post-natal period, which is evident as pancreatic alpha cells have a poor response to parenteral glucose prior to parturition [5]. Both alpha and beta pancreatic cells undergo developmental changes during the first ten days of life in newborn pony foals [6]. Since development of the endocrine pancreas continues into the post-natal period and there is transient reduced insulin action due to increased cortisol concentrations [7–9], it has been suggested that neonatal foals are insulin resistant in the first 24 hours post-partum [9].

Hypoglycemia is commonly encountered in the equine neonate, particularly during states of critical illness and septicemia [10, 11], supporting the assumption that maturation of the energy endocrine system may be delayed in the neonate [12]. Equine neonates often undergo periods of hypoglycemia due to an inability to nurse, intolerance to enteral nutrition, or lack of readily available energy stores [3, 4, 9]. Hypoglycemia can be treated with enteral or parenteral nutrition. However, a frequent complication of parenteral nutrition in foals and horses is hyperglycemia, which is associated with a worse outcome in critically ill neonatal foals [10]. In infants, hyperglycemia is also a poor prognostic indicator that is thought to be a consequence of the systemic inflammatory response and can lead to endothelial cell dysfunction [13].

There are many methods used to evaluate insulin and glucose dynamics in the horse, some of which have been performed in healthy and critically ill neonatal foals [14]. Clinically, non-specific indicators of insulin resistance, such as basal glucose and insulin concentrations, glucose tolerance tests, and proxy measurements, can be performed [15]. Although these methods are easy to perform, minimally invasive, and financially feasible, they only provide estimates of insulin dysregulation [16]. Dynamic, quantitative tests, which are considered the gold standard in measuring insulin and glucose dynamics, include the insulin-modified frequently sampled intravenous glucose tolerance test (I-FSIGTT) with minimal model analysis and the euglycemic-hyperinsulinemic clamp (EHC). The I-FSIGTT with minimal model analysis has been evaluated in the horse previously [17–22]. Toth et al. [21] optimized this protocol by decreasing the dextrose dose to reduce urinary glucose loss and provide more accurate results based on minimal model parameters.

To our knowledge, few studies have used dynamic testing methods with minimal model analysis to evaluate energy regulation in the neonatal foal [6, 23]. The objective of this study was to compare the I-FSIGTT with minimal model analysis and proxy measurements of insulin sensitivity between healthy neonatal foals and horses to further enhance our understanding of age-related insulin and glucose dynamics, and therefore, energy homeostasis. We hypothesized that healthy neonatal foals will exhibit higher insulin and glucose responses to intravenous dextrose compared to the response in horses, and that this may be due to decreased glucose clearance in response to insulin, or physiologic insulin resistance occurring during the neonatal period.

## 2. Materials and methods

### 2.1 Animals

Twelve neonatal Standardbred foals of 24 to 60 hours of age and owned by a private breeding farm (Midland Acres Inc, Bloomingburg, Ohio, USA) were enrolled. All foals were determined clinically healthy based on physical examination, normal hemogram, and serum immunoglobulin G (IgG) concentration > 800 mg/dL. Eight adult horses, 3 to 14 years of age, were also enrolled. All horses were determined clinically healthy based on physical examination, blood work, were under a regular vaccination and deworming program, and had no recent history of disease, including no evidence of laminitis. Breeds included Quarter Horse (n = 3), Thoroughbred (n = 2), and Standardbred (n = 3). Horses were owned by the university (n = 5) and a private breeding farm (n = 3; Midland Acres Inc). Foals and horses were excluded from the study if their baseline insulin concentration was > 20 μIU/ml [24]. Please see S5 Table for specific subject information.

### 2.2 Experimental design

The I-FSIGTT was performed on all neonatal foals and horses. Briefly, neonatal foals were muzzled one hour before initiation of the I-FSIGTT and horses were muzzled for 12 hours before initiation of the I-FSIGTT. None of the animals were allowed to feed/nurse during the testing protocol. An intravenous catheter (Surflo IV Catheter 14G, Terumo Medical, Somerset, New Jersey, USA) was placed one hour prior to testing. Baseline blood samples were drawn 60 minutes and immediately prior (time 0) to test initiation. A 50% dextrose solution (300 mg/kg) (VetOne, MWI Animal Health, Boise, Idaho, USA) was administered IV (time 0). The dose of dextrose administered was based on previous data from our laboratory [25], as well as preliminary data showing that this dose of dextrose (300 mg/kg) resulted in a tolerable level of hypoglycemia (without clinical signs) in response to insulin (Unpublished data, LDH & HMK). Blood samples were collected at -60, 0, 2, 5, 7, 10, 15, 19, 22, 25, 30, 35, 40, 50, 60, 75, 90, 120, 150, and 180 minutes relative to administration of dextrose. Regular insulin (Humulin R, Lilly USA, Indianapolis, Indiana, USA) at a dose of 0.02 IU/kg was diluted in 3 mL sterile saline and administered IV 20 minutes after administration of dextrose. After 180 minutes, animals were unmuzzled, and allowed to feed/nurse ad libitum.

Blood samples were placed in serum tubes and pre-chilled EDTA tubes containing aprotinin (GoldBio, St. Louis, Missouri, USA) and diprotin A (Bachem, Torrance, California, USA). Serum samples were allowed to clot at room temperature for one hour. Plasma and serum samples were centrifuged at 1000 g for 15 minutes at 4°C and were then aliquoted and stored at -80°C until analysis.

This study was approved by The Ohio State University Institutional Animal Care and Use Committee and adhered to the principles of humane treatment of animals in veterinary clinical investigations as stated by the American College of Veterinary Internal Medicine and National Institute of Health guidelines.

### 2.3 Sample analysis

Whole blood glucose concentrations were determined immediately throughout the study protocol using a hand-held glucometer (AlphaTRAK^®^, Zoetis Inc., Parsippany, New Jersey, USA) validated for use in horses [26]. Serum insulin concentrations were measured using a human-specific ELISA (MP Biomedicals, Solon, Ohio, USA) previously used by our laboratory for equine samples [25].

### 2.4 Assessment of insulin sensitivity

MinMod Millennium software [17] was used to create graphical representations of the I-FSIGTT in neonatal foals and horses, as well as calculate parameters of insulin and glucose dynamics (insulin sensitivity [SI], glucose effectiveness [Sg], acute insulin response to glucose [AIRg], disposition index [DI]). Definitions for minimal model parameters can be found in Table 1. Area under the curve (AUC) for insulin and glucose was determined by the trapezoidal method using statistical software (GraphPad Prism 8.0, San Diego, California, USA). Proxy measurements (homeostasis model assessment of beta cell function [HOMA-BC%], homeostasis model assessment of insulin resistance [HOMA-IR], quantitative insulin sensitivity check index [QUICKI], reciprocal square root of insulin [RISQI], and modified insulin to glucose ratio [MIRG]) were calculated using fasted baseline blood glucose and serum insulin concentrations [15] (Table 2). For all HOMA indices, blood glucose concentrations were converted from mg/dL to mmol/L.

**Table 1:** Minimal model parameters measured and definitions.

Minimal Model Parameter	Definition
**Insulin Sensitivity (SI)**	Capacity for insulin to promote disposal of glucose and inhibit endogenous glucose production
**Glucose Effectiveness (Sg)**	Ability of glucose to mediate its own disposal
**Acute Insulin Response to Glucose (AIRg)**	First phase insulin response to glucose
**Disposition Index (DI)**	Ability of the pancreatic islet cells to secrete insulin normalized to degrees of insulin resistance

**Table 2:** Formulas and measured effects for proxy measurements of insulin resistance.

Proxy Measurement	Calculation	Effect Measured
HOMA IR	(glucose × insulin)/22.5	Insulin Resistance
RISQI	insulin-0.5 = 1√insulin	Insulin Sensitivity
QUICKI	1/(log[insulin] + log[glucose])	
MIRG	(800–0.3 × [insulin − 50]2)/(glucose − 30)	Beta Cell Function
HOMA-BC%	(20 × insulin)/(glucose − 63)	

HOMA-IR, homeostasis model assessment of insulin resistance; HOMA-BC%, % homeostasis model assessment of beta function; RISQI, reciprocal of the square root of insulin; QUICKI, quantitative insulin sensitivity check index; MIRG, modified insulin to glucose ratio.

### 2.5 Data analysis

Data were tested for normality with the Shapiro-Wilk test and were not normally distributed. Descriptive statistics are reported as median and interquartile range (IQR). Minimal model parameters and proxy measurements were compared between foals and horses using Mann-Whitney U test. No correction for multiple comparisons was applied since these parameters describe different aspects of glucose and insulin homeostasis. Spearman rank correlation was performed to investigate a potential relationship between SI and foal age in hours. All time points for blood glucose and insulin concentrations were compared to baseline using Friedman’s test. Dunn’s post hoc test was used for multiple comparisons. AUC data were normally distributed and presented as mean and 95% confidence interval. AUC_insulin_ and AUC_glucose_ were compared between foals and horses using an independent t-test. Statistical analysis was performed using SPSS 26 (IBM^®^, Armonk, New York, USA) and Prism 8.0 (GraphPad Software Inc., San Diego, California, USA). Statistical significance was set at P < 0.05.

## 3. Results

### 3.1 Glucose

**Fig 1:**
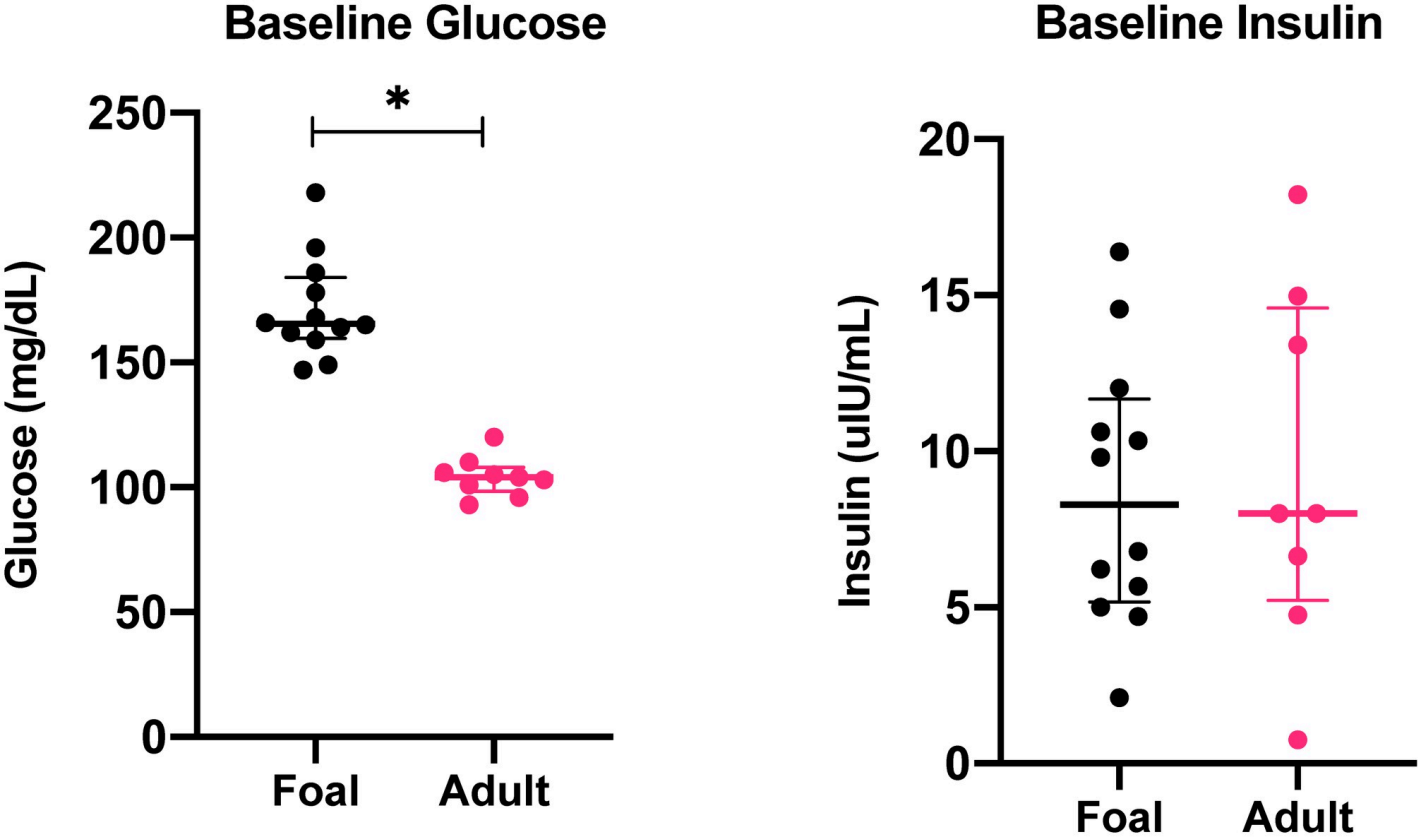
Baseline (time 0) blood glucose and serum insulin concentrations (median and IQR) in healthy foals (black circles) and horses (pink squares). * indicates statistically significant difference between foals and horses. IQR, interquartile range.

**Fig 2:**
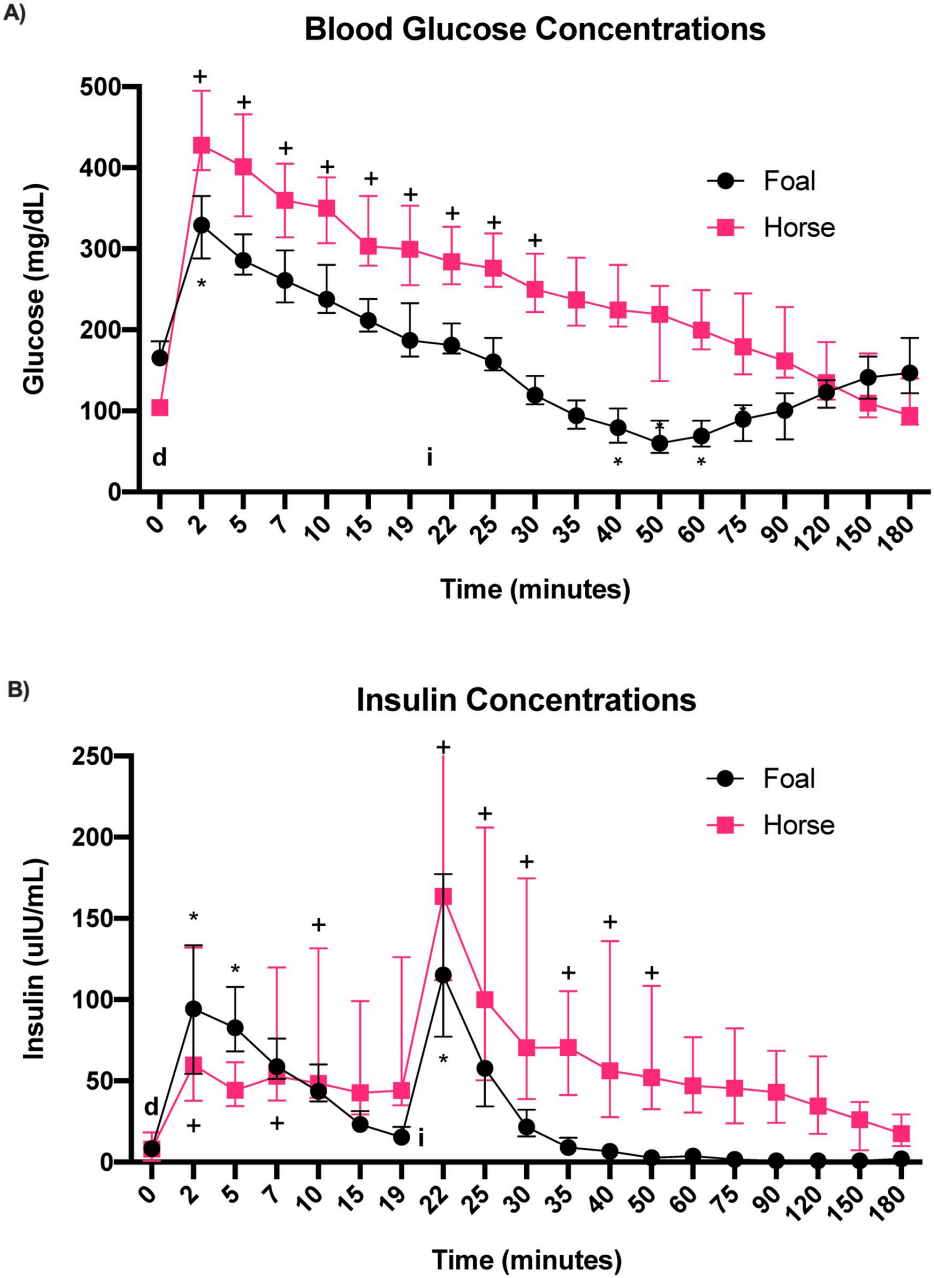
Blood glucose and serum insulin (median and IQR) concentrations during the I-FSIGTT; A) glucose concentrations in foals (black circles) and horses (pink squares), B) insulin concentrations in foals (black circles) and horses (pink squares). Lower case d at time 0 indicates dextrose administration (300 mg/kg, IV) and lower case i at 20 minutes indicates insulin administration. * and + indicates statistically significant difference from time 0 for foals and horses, respectively. IQR, interquartile range; I-FSIGTT, insulin-modified frequently sampled intravenous glucose tolerance test.

Median baseline glucose concentrations (Fig 1; S1 Table) were significantly higher in neonatal foals (166 mg/dL [160–184]) compared to horses (104 mg/dL [98.5–120]); (Mann-Whitney U, p < 0.0001). In foals, glucose concentrations increased significantly immediately (2 minutes) after dextrose administration (0 minutes; F = 207.2; adjusted p-value = 0.02). Subsequently, glucose concentrations decreased significantly 20–55 minutes after insulin administration (40, 50, 60 and 75 minutes after dextrose administration; Fig 2; p = 0.0084, 0.0002, 0.0006, 0.0369, respectively). In horses, glucose concentrations increased significantly compared to baseline (0 minutes) from 2 to 30 minutes (Fig 2; F = 174.2; adjusted p-value < 0.0001 [2, 5, 7, 10, 15, 19, 22, and 25 minutes], p = 0.0114 [30 minutes]) and continually decreased after insulin administration. This is in contrast to foals, in which glucose concentrations began to rebound at 60 minutes (40 minutes after insulin administration; p < 0.001). AUC_glucose_ (Table 3) was significantly higher in horses (34,068 mg/dL·min [30,927–37,209]) compared to foals (23,138 mg/dL·min [20,573–25,703]); (t = 5.282; df = 18, p < 0.0001).

**Table 3:** Area under the curve (mean and 95% confidence interval) for insulin and glucose in foals and horses.

	Glucose (mg/dL·min)		Insulin (μIU/mL·min)	
	AUC	95% CI	AUC	95% CI
**Foal**	23,138*	20,573–25,703	2,100*	1,758–2,441
**Horse**	34,068*	30,927–37,209	8,472*	6,980–9,964

* indicates statistically significant difference between horses and foals (t = 5.828; df = 18 p < 0.0001[glucose] t = 9.81; df = 18; p < 0.0001 [insulin]).

### 3.2 Insulin

Median baseline serum insulin concentrations (Fig 2; S2 Table) were 8.3 μIU/mL (5.2–11.7) for foals and 8 μIU/mL (5.2–14.6) and horses, which were not significantly different between groups (Mann-Whitney U; p = 0.7774). In neonatal foals, serum insulin concentrations were significantly higher compared to baseline at 2 and 5 minutes after dextrose administration (F = 201.1; p = 0.0013 [2 minutes]; p = 0.0023 [5 minutes], and 2 minutes after insulin administration (Fig 2; p < 0.001). In horses, serum insulin concentrations were significantly higher than baseline (time 0) at times 2, 7, 10 minutes after dextrose administration (F = 107.1; p < 0.001, p = 0.0391, p = 0.0024, p = 0.0034, respectively), and 2 to 30 minutes after insulin administration (Fig 2; p < 0.001 [2–15 minutes after insulin]; p = 0.002 [20 minutes]; p = 0.0155 [30 minutes post-insulin]). The AUC_insulin_ (Table 3) was significantly higher in horses (8,472 μIU/mL·min [6,980–9,964]) compared to foals (2,100 μIU/mL·min [1,758–2,441]); (t = 9.81, df = 18, p < 0.0001).

### 3.3 Minimal model analysis

**Fig 3:**
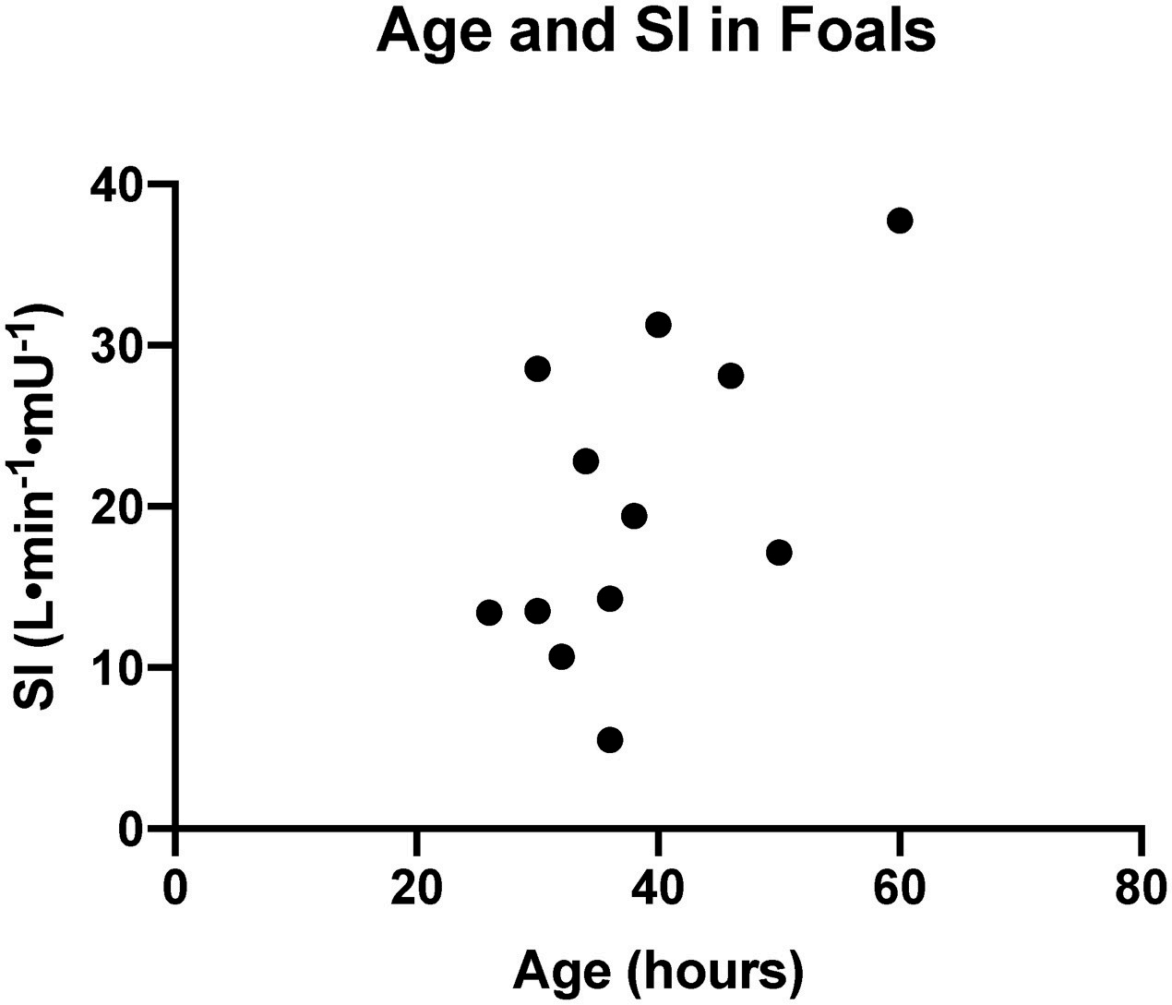
Spearman rank correlation between foal age in hours and SI (r = 0.512, p = 0.091). SI, insulin sensitivity.

Minimal model parameters were not different between horses on different farms (Mann-Whitney U, p = 0.7857, p = 0.5714, p = 0.39329, p = 0.7857 for SI, AIRg, Sg, and DI, respectively) and were combined into one group. Insulin sensitivity (SI) was significantly higher in foals (18.3 L·min^-1^· μIU^-1^ [13.4–28.4]) compared to horses (0.9 L·min^-1^· μIU^-1^ [0.5–1.1]); (p < 0.0001). Correlation between foal age in hours and SI was not significant but had a positive trend (r = 0.512; p = 0.091) (Fig 3). Disposition index (DI) was also significantly higher in foals (1.2 × 10^4^ [0.8–1.4]) compared to horses (0.04 × 10^4^ [0.02–0.07]); (p < 0.0001). The AIRg values were not different (p = 0.2991) between foals (605 μIU/L·min^-1^ [452–804]) and horses (370 μIU/L·min^-1^ [330–650]). Glucose effectiveness (Sg) values were also not different (p = 0.0556) between foals (0.02 min^-1^ [0.014–0.021] and horses (0.01 min^-1^ [0.008–0.017]) (Table 4; S3 Table).

**Table 4:** Minimal model parameters and proxy measurements of insulin resistance in foals and horses (median and IQR).

	Foal	Horse
**SI (L·min** ^**-1**^ **·μIU** ^**-1**^ **)**	18.3 (13.4–28.4)*	0.9 (0.5–1.1)*
**Sg (min** ^**-1**^ **)**	0.02 (0.014–0.021)	0.01 (0.008–0.017)
**AIRg (μIU/L·min** ^**-1**^ **)**	605 (452–804)	370 (330–650)
**DI (× 10** ^**4**^ **)**	1.2 (0.8–1.4)*	0.04 (0.02–0.07)*
**HOMA-IR (mmol/L·μIU/mL)**	3.58 (2.11–5.13)	1.97 (1.38–3.96)
**HOMA-BC% (%)**	23.2 (17.8–42.2)*	78.4 (43.0–116.0)*
**RISQI (1/√μIU/mL)**	0.32 (0.29–0.42)	0.35 (0.26–0.39)
**QUICKI (mg/dL·μIU/mL)**	0.31 (0.3–0.34)	0.34 (0.31–0.35)
**MIRG ([μIU/mL]** ^**2**^ **·dL/mg)**	1.72 (1.43–2.68)*	3.91 (2.57–5.37)*

* indicates statistically significant difference between foals and horses.

IQR, interquartile range; SI, insulin sensitivity; Sg, glucose effectiveness; AIRg, acute insulin response to glucose; DI, disposition index; HOMA-IR, homeostasis model assessment of insulin resistance; HOMA-BC%, % homeostasis model assessment of beta function; RISQI, reciprocal of the square root of insulin; QUICKI, quantitative insulin sensitivity check index; MIRG, modified insulin to glucose ratio.

### 3.4 Proxy measurements

HOMA-BC% was higher in horses (78.4% [43.0–116.0]) compared to foals (23.2% [17.8–42.2]); (p = 0.0096). MIRG was lower in foals (1.72 μIU_insulin_^2^/10·mg_glucose_ [1.43–2.68]) compared to horses (3.91 μIU_insulin_^2^/10·L·mg_glucose_ [2.57–5.37]); (p = 0.0092). HOMA-IR, QUICKI, and RISQI were not significantly different between foals and horses (Mann-Whitney U; p = 0.0783, p = 0.1507, and p = 0.6429, respectively) (Table 4; S4 Table).

## 4. Discussion

The neonatal foal undergoes several physiologic changes at birth in order to survive the transition to extra-uterine life. Many of the changes occurring during this transition, such as glucoregulation, are not present prior to birth but are required for survival [27]. In this study, we showed that compared to horses, healthy neonatal foals are more insulin sensitive, with insulin having a higher capacity to promote glucose disposal and inhibit endogenous glucose production. This finding was unexpected, as neonatal foals have been considered to be physiologically insulin resistant as part of their delayed endocrine maturation and perinatal increases in cortisol concentrations [6–8].

Disposition index was elevated in foals compared to adults, suggesting that the endocrine pancreas in newborn foals has a high insulin secretory capacity in order to favor energy conservation. This aligns with higher SI values in foals, as DI is the product of SI and AIRg. It is possible that foals adapted to produce insulin to preserve energy during the critical transition into extrauterine life, but also as a protective mechanism against insulin resistance.

Although SI and DI values were higher in foals compared to horses, Sg values were not different between groups. AIRg values trended higher in foals compared to horses but were not significantly different, further suggesting that foals may have a higher capacity to secrete insulin in response to hyperglycemia. Since neonatal foals have minimal energy stores at birth compared to other species [28, 29], perhaps our findings reflect an evolutionary adaptation to conserve energy during the transition to extrauterine life.

Effects of age on insulin and glucose dynamics have been reported in the literature in neonatal foals [6, 9, 30], and results are somewhat conflicting. Spontaneously delivered and healthy neonatal foals have shown no changes in glucose or insulin dynamics in the first days of life [6, 30] in some studies; whereas, other studies have reported younger foals (1 day of age) have decreased glucose clearance compared with older foals, suggesting a degree of insulin resistance in the early neonatal period [9]. Smyth et al. [31] suggested pancreatic beta cells may mature with age, as younger foals (1 day, 1 week, 1 month of age) had lower insulin concentrations compared to foals at 3 months of age. Based on findings from our study, where SI was moderately and positively correlated with increasing age (Fig 3), we suspect the neonatal foal does undergo maturational changes within the endocrine pancreas during the early neonatal period. Although this trend was non-significant, perhaps improvement in correlation would be observed with more foals examined.

Proxy measurements of insulin sensitivity are commonly used in human medicine to estimate insulin dysregulation. In the horse, proxy measurements have also been used as indicators of insulin resistance [18, 32, 33]. The QUICKI, RISQI, and HOMA-IR serve as estimates of insulin sensitivity, while MIRG and HOMA-BC% are estimates of pancreatic beta cell function or insulin secretion from the pancreas. When proxy measurements of insulin sensitivity and insulin secretion were compared between foals and horses in our study, foals were found to have similar insulin sensitivity values to adults, with no differences in QUICKI, HOMA-IR, or RISQI. Although foals had similar insulin sensitivities compared to horses, HOMA-BC% and MIRG values were higher in horses compared to foals, suggesting that the foal may have decreased pancreatic insulin secretory ability relative to elevations in blood glucose concentrations. This further supports that neonatal foals may be more insulin sensitive than horses, which may be to compensate for reduced pancreatic insulin secretory ability. This finding is also supported by higher AUC_insulin_ values and lower baseline glucose concentrations observed in horses compared to foals, considering administration of the same dose of dextrose. Based on the results of our study, we emphasize that the neonatal foal may be more insulin sensitive than previously reported [6, 9, 12, 34].

Our results also emphasize the important differences in testing strategies employed when assessing insulin dysregulation in the equid, as results concluded from the insulin secretory ability from the pancreas based on I-FSIGTT and proxy measurements are conflicting. Results from minimal model analysis indicate that foals have increased insulin secretory capacity compared to horses. However, proxy measurements indicate that horses may have increased secretory ability from the pancreas compared to foals. Based on our contradicting results, proxy measurements of insulin sensitivity may not be accurate for assessment of insulin dysregulation in the neonatal foal based on foals’ physiologically higher basal glucose concentrations.

Our study revealed that baseline glucose concentrations in foals were significantly higher than in horses. The baseline blood glucose concentrations reported in our study from healthy neonatal foals are also higher than reference values commonly used for horses [35, 36] and foals in the clinical setting. Hyperglycemia, as a result of parenteral nutrition, in critically ill foals and infants has been associated with a poor outcome [13, 37]. Tight glycemic control based on insulin administration has been associated with decreased mortality in critically ill human patients [38]. However, hypoglycemia occurring in hospitalized human patients under treatment protocols for tight glycemic control has also been demonstrated to increase the risk of mortality [39]. The higher baseline glucose concentrations measured in the healthy foals of this study calls into question whether the goals for glycemic control in the equine neonate should be re-examined.

Critically ill neonatal foals are prone to hypoglycemia, which can be secondary to reduced milk intake, little glycogen storage after birth, and sepsis-mediated impaired gluconeogenesis [14, 28]. Based on this study and previous studies performed by our laboratory [25, 40], healthy equine neonates with no access to nursing do not develop hypoglycemia for at least 4 hours of milk restriction. Published reference ranges for glucose in the neonatal foal are higher than those used for adults [36, 41, 42], and the results of this study further solidify that this should be taken into account in the clinical management of the neonatal foal [41, 42]. The question of whether the use of tight or liberal glycemic control is more beneficial in the treatment of the critically ill neonate continues to remain controversial, and additional investigation is warranted.

Neonatal foals undergo periods of hypoglycemia as a consequence of little energy stores at birth and inadequate glycemic control in the post-natal period [10, 28]. During our study, foals exhibited mild to moderate hypoglycemia after administration of insulin (0.02 IU/kg IV), with the lowest blood glucose concentration observed being 40 mg/dL approximately 30 minutes after insulin administration. Of interest, none of the foals showed clinical signs consistent with hypoglycemia [43]. During the study period, glucose concentrations began to rebound 40 minutes after insulin administration, reaching baseline values at 180 minutes (Fig 2). These changes in glucose concentrations were not observed in horses, which is likely a result of more efficient gluconeogenesis. Adult horses also did not have as robust of a response to insulin compared to foals and did not become hypoglycemic. These results contradict previous beliefs regarding insulin resistance in the neonatal foal, and support the idea that healthy neonatal foals are able to mount appropriate responses to regulate blood glucose during the post-natal period despite reports in the literature regarding inadequate glycemic control in the post-natal period [10, 28].

A limitation of our study was that some of the horses had lower SI values when compared to previous studies [18, 24, 32, 44, 45]. This could be a result of age, diet, breed [19, 44, 45], or the insulin assay used [46–48]. The horses in our study were housed in two separate locations under different management practices and diets. Approximately half of the adults were adapted to a primarily forage/fiber diet, while the other half were adapted to non-structural carbohydrate meals and pasture. High starch diets are associated with lower insulin sensitivities in weanlings and horses [49–52], and could be a factor to explain lower SI values in the horses of our study. However, this could also be a result of management practices and the environment, which may not be comparable between different studies. However, minimal model parameters (SI, Sg, AIRg, and DI) were not different for horses when grouped by farm in our study. The same glucose and insulin doses were used in foals and adults to provide accurate comparisons and have been used previously [32, 44, 45]. Although adult SI values were low in our study, the values observed in foals were considerably higher than SI values reported in adults elsewhere [18–21, 32, 44, 45]. Horses in our study were also fasted prior to and during the protocol, which has the potential to alter the action of insulin by increasing activity of the hypothalamic-pituitary-adrenal axis [53], and could have affected insulin sensitivity values in our study.

Our study sought to evaluate glucose and insulin dynamics in healthy newborn foals and compare values to healthy horses. Our results challenge concepts presented in the literature stating that newborn foals are insulin resistant secondary to endocrine modifications that occur in the perinatal period. In fact, we showed that the neonatal foal is insulin sensitive in the first days of life. Neonatal foals may also have mechanisms for maintaining euglycemia in order to prevent hypoglycemia during the postnatal period. Our findings may indicate that evolutionary adaptations exist during the neonatal period in order to successfully transition to extrauterine life.

## Supporting information

S1 Table Blood glucose concentrations. (XLSX)

S2 Table Insulin concentrations. (XLSX)

S3 Table Minimal model parameters. (XLSX)

S4 Table Proxy measurements. (XLSX)

S5 Table Subject information. (XLSX)
